# Malay Version of Exercise Self-Efficacy: A Confirmatory Analysis among Malaysians with Type 2 Diabetes Mellitus

**DOI:** 10.3390/ijerph17030922

**Published:** 2020-02-02

**Authors:** Aizuddin Hidrus, Yee Cheng Kueh, Bachok Norsa’adah, Garry Kuan

**Affiliations:** 1 Unit of Biostatistics and Research Methodology, School of Medical Sciences, Universiti Sains Malaysia, Kubang Kerian 16150, Malaysia; aizuddinh88@gmail.com (A.H.); norsaadah@usm.my (B.N.); 2 Community and Family Medicine Department, Faculty of Medicine and Health Science, Universiti Malaysia Sabah, Kota Kinabalu 88400, Malaysia; 3 Exercise and Sports Science, School of Health Sciences, Universiti Sains Malaysia, Kubang Kerian 16150, Malaysia; garry@usm.my; 4 Department of Life Sciences, Brunel University, London UB8 3PH, UK

**Keywords:** validity, reliability, confirmatory factor analysis, diabetes, psychometric evaluation

## Abstract

Exercise self-efficacy (ESE) is one of the psychological constructs in the Transtheoretical Model (TTM). The objective of the present study is to assess the validity and reliability of the Malay version of Exercise self-efficacy scale (ESE-M) among Malaysians with type 2 diabetes mellitus (T2DM). A cross-sectional study design with convenience sampling method using a self-administered questionnaire was carried out. Participants were invited to complete the ESE-M with 18 items. Confirmatory factor analysis (CFA) was conducted and composite reliability (CR) was computed using Mplus 8. A total of 331 Malaysians with T2DM with a mean age of 63 years old (Standard Deviation = 0.57) completed the questionnaire. Most of the participants were male (52%) and Malay (89.4%). Two initial CFA models (single factor and three factors) of ESE-M scale were tested and they did not fit to the data well. Several re-specifications of the models were conducted. The final model for the ESE-M showed improvement on the value of model fit indices for the single factor model (comparative fit index (CFI) = 0.952, Tucker and Lewis index (TLI) = 0.938, standardised root mean square (SRMR) = 0.044, root mean square error of approximation (RMSEA) = 0.054) and three factors model (CFI = 0.891, TLI = 0.863, SRMR = 0.049, RMSEA = 0.081). The CR for the self-efficacy factor was 0.921 (single factor), while CR for internal feelings, competing demands and situational (three factors) were 0.762, 0.818 and 0.864, respectively. The final model of single factor ESE-M showed better fit to the data compared to the three factors ESE-M. This indicated that the single factor ESE-M is more suitable to be adopted for future study among Malaysians with T2DM.

## 1. Introduction

The World Health Organization (WHO) defined physical activity (PA) as “any bodily movement produced by skeletal muscles that require energy expenditure” [1]. Walking, cycling or any participation in sports are examples of the moderate intensity PA that can be done regularly and beneficial for health. For many years, PA and exercise had been empirically accepted by clinicians and researchers as a regimen to improve the health status of patients with any kind of diseases. A systematic review conducted by Anderson et al. [2] found that coronary heart disease patients who were given exercise training (intervention group) showed a decrease in cardiovascular mortality, hospital admissions and improvement in health-related quality of life compared to the non-exercise control group.

According to the report of Malaysia National Health and Mortality Survey (NHMS) in 2015 [3], physical inactivity is the fourth worldwide mortality risk factors. It is noted that risk of death increases 20–30% among people who physically inactive. Other than cardiovascular diseases (CVD), physical inactivity is also one of the recognised crucial risk factors for type 2 diabetes mellitus (T2DM). In NHMS, they categorised PA into three groups, inactive (insufficiently active people), minimally active (sufficiently active people) and HEPA (health enhancing physical activity) active (people who are doing more than the sufficiently/minimum recommended PA). They reported that 66.5% of Malaysian adults are physically active. Nevertheless, although more than 50% of Malaysian adults are physically active, the report indicated that there are only 25.4% of Malaysian adults that count as HEPA active. The remaining 41.1% are categorised as minimally active.

Diabetes mellitus is one of the most common non-communicable diseases (NCD) in the world other than CVD, cancers and respiratory diseases. WHO reported that T2DM is more commonly diagnosed compared to type 1 diabetes mellitus. In Malaysia, T2DM became one of the most burdening NCDs that needs to be handled not only by the doctors and healthcare staffs, but also by the patients themselves. Having the routine medications (as prescribed by the doctors) on time, taking minimal amount of daily sugar and being physically active are the patients’ responsibility. However, a survey reported that 54% of diabetes patients in Malaysia are physically inactive [4,5]. Excuses such as, “not having enough time to do physical activity”, “not feeling well for exercise” and “focusing on other things to do” are the most common excuses given by our community including T2DM patients.

Self-efficacy is a person’s belief in their potential of doing and achieving the given goals and targets that could give them high benefits by vanquishing all the obstacles that came their way. A person with high self-efficacy is perceived to be more excited while carrying out the given tasks by giving more endeavours to achieve the goals [6]. Despite the given tasks and goals that are challenging and come with obstacles, a person with high self-efficacy takes the obstacles as tests and is not afraid of failure in order to achieve success [7]. Physical self-efficacy is found as a solid predictor for a person in participating and maintaining the act of PA [8]. Moreover, self-efficacy also appeared as a major predictor for the choosing types of PA, level of endeavours for the chosen PA and on how to handle the obstacles while executing the chosen PA [9]. 

In the Transtheoretical Model (TTM), self-efficacy is one of the psychological constructs of the model. Exercise self-efficacy scale (ESE) was developed by Bandura [6]. The scale is to measure the level of a person’s self-efficacy towards PA. According to Resnick and Jenkins [10], ESE scale was the first validated and reliable tool as well as being considered as the most commonly adopted scale in measuring an individual self-efficacy. Since then, researchers have been actively translating the ESE scale into their own language including, Korean [11], Chinese [12], Swedish [13] and Malay [14]. Until now, the scale has been adopted on different population such as, African American and Latino elderly in United States of America [15], diabetes patients in Taiwan [16] and Swedish people [17]. On the other hand, the Malay version of the self-efficacy for exercise scale [14] was applied on undergraduate students. All undergraduate students should have a high level of intelligence with good understanding to answer such questionnaires. In contrast to the present study, we were examining the validity and reliability of the Malay version of exercise self-efficacy (ESE-M) scale among Malaysians with T2DM. People with T2DM are varied in different age groups, have different level of education and socioeconomic backgrounds and have diverse ways of understanding questionnaires. Hence, it is necessary to determine the validity and reliability of the ESE-M in the present study before it is utilised in measuring the self-efficacy of PA among people with T2DM in future study. Further, intervention should be implemented in corresponding to the measured self-efficacy level of T2DM patients, in order to motivate them to initiate or maintain of performing PA.

## 2. Materials and Methods

### 2.1. Study Design and Population

Data collection was conducted in the Hospital Universiti Sains Malaysia (HUSM) Kubang Kerian, Kelantan, Malaysia. The data collection was conducted between October 2018 and December 2018. A cross-sectional study design with convenience non-probability sampling method was carried out among the adults (18 years old and above) with T2DM. People with T2DM who were treated in HUSM were approached to participate in the present study. They were explained about the purpose and benefits of the present study by the researcher. Those who agreed to volunteer were asked to complete the consent form before answering the self-administered questionnaire. They took between 10–15 minutes to complete the questionnaire in HUSM.

### 2.2. Inclusion and Exclusion Criteria of Participants

Participants that included in the present study are those who were clinically diagnosed with T2DM for at least a year, Malaysian nationality, age of 18 years old and above, able to read and understand Malay language, able to fill in the given questionnaire and understand the information that was explained by the researcher. People who were diagnosed with T2DM, as well as having mental illness were excluded from the present study.

### 2.3. Sample Size Determination

Kline [18] suggested that an acceptable sample size for studies using structural equation modelling (SEM) is about 200 cases. This number corresponds to the approximate median sample size in surveys of published article by Shah and Goldstein [19] of 93 articles in management science journals. Confirmatory factor analysis (CFA) is one of the components of SEM. Therefore, the sample for the present study of 331 people with T2DM is considered adequate.

### 2.4. Instrument

The self-administered questionnaire consists of two sections, the demographic and the ESE-M. As for the demographic section, information such as age (years), gender, ethnicity, diabetes period, BMI and HbA1c of the patients were collected through the section. 

Malay version of Exercise Self-efficacy Malay (ESE-M). The original English version was developed by Bandura [8] with a single factor of self-efficacy. The scale was translated into Korean language and revised into three factors (internal feelings with seven items, competing demands with five items and situational with six items) and 18 items which was adopted by Shing, Jang and Pender [11], Kim [20] and Kosma [21] in their studies. It is developed with a 5-point Likert scale ranging from “1 = cannot do” to “5 = certain can do”. The original English version of the ESE scale was translated into the Malay language using the forward and backward procedures and based on Brislin method were outlined in previous studies [14,22]. The Malay version of the scale had been validated among university undergraduates [14]. 

Due to the discrepancy of the results found in the previous studies [8,11,14] in regards to the factor of the structure of exercise self-efficacy scale, we decided to assess the validity of both versions of factor structures: single factor and three factors, to confirm the most suitable factor structure of ESE-M for the Malaysian with T2DM. Thus, future researchers could decide whether they should interpret ESE-M based on one factor (i.e., self-efficacy) or three factors (i.e., internal feelings, competing demands, situational) when using it on people with T2DM.

### 2.5. Ethical Consideration

The study obtained approval from USM Human Research Ethics Committee (USM/JEPeM/18040201) and was conducted in accordance with the guidelines of the International Declaration of Helsinki. Participants were informed that their participation was voluntary and they may withdraw at any time without any loss or penalty. Participants who volunteered to participate in the study completed the questionnaire, which included the demographic sheet and the ESE-M. Participants’ written informed consent were obtained prior to the completion of the questionnaire. 

### 2.6. Data Analysis

Demographic data of participants were presented by descriptive information. For categorical variables, they were presented by frequencies and percentage (%), whereas for numerical variables, they were presented by mean and standard deviation (SD). 

Data analysis of confirmatory factor analysis (CFA) was performed by using statistical software Mplus 8 to assess the validity of two hypothesized ESE-M measurement models (single factor of self-efficacy and three factors of internal feelings, competing demands and situational) with 18 items. In the CFA analysis, all the 18 items were treated as observed variables and the factor(s) was considered as a latent variable(s). 

The data were checked for multivariate normality, and results indicated that the data did not meet the assumption, based on Mardia multivariate skew (*p* < 0.001) and kurtosis (*p* < 0.001) tests. Therefore, for the subsequent CFA, the robust maximum likelihood estimator (MLR) was utilised, as this is robust to non-normality [23,24,25]. Researchers had suggested presenting more than one fit index in order to show the validity of the questionnaire [26]. The fit indices and its acceptable threshold value are as follows: the comparative fit index (CFI) and Tucker and Lewis index (TLI) with the desired value of more than 0.92; the root mean square error of approximation (RMSEA) with the desired value of less than 0.08; probability RMSEA with the desired value of more than 0.07; and the standardised root mean square (SRMR) with the desired value of less than 0.08 [27]. 

An item with factor loading less than 0.4 was treated as a problematic item [28,29] and subjected for removal after adequate theoretical support was carried out. The CFA modification index (MI) was inspected during model re-specification to obtain the best fit measurement models. The model re-specification included adding correlation between items’ residuals within the same factor. The model was re-specified after the authors obtained adequate theoretical support.

Reliability of the final ESE-M measurement model was assessed using composite reliability (CR) based on Raykov’s method by using Mplus [30]. The minimum acceptable range of CR is 0.60 and above [31].

## 3. Results

### 3.1. Demographic Characteristics

A total of 331 people with T2DM in HUSM were involved in the present study (see Table 1). Mean age of the participants was 63 (0.57). Out of the total sample size, 172 (52%) of them were male and 159 (48%) were female. Malay participants were the majority ethnic with a total number of 296 (89.4%), followed by 25 (7.6%) Chinese, 6 (1.8%) Indian and 4 (1.2%) other. The mean of diabetes duration since diagnosis of the participants was 19 (SD = 0.62) years. While for BMI and HbA1c, the means were 27.27 kg/m^2^ (SD = 0.28) and 76.9 mmol/mol (SD = 1.33), respectively. 

### 3.2. Confirmatory Factor Analysis

#### 3.2.1. ESE-M with Single Factor

**Figure 1:**
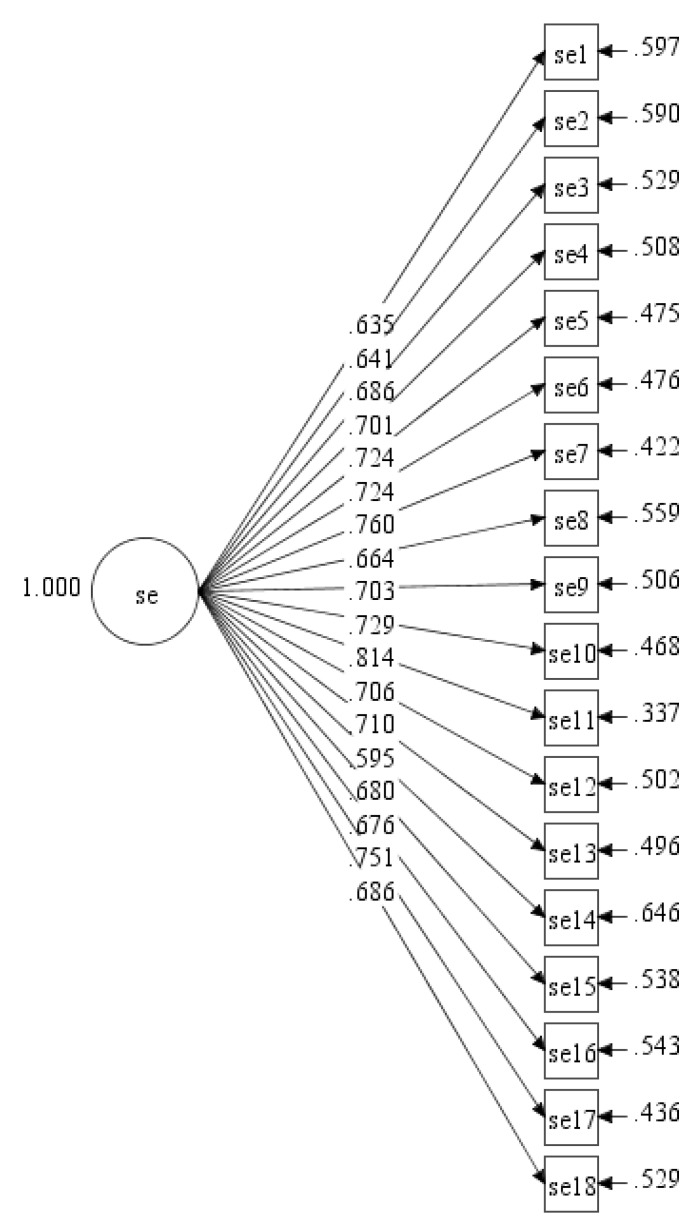
Initial hypothesised Malay version of Exercise self-efficacy scale (ESE-M) measurement model with single factor (se = self-efficacy).

It was aforementioned that the earliest version of the scale was developed with only the single factor of self-efficacy by Bandura. In the first (Initial) tested hypothesised ESE-M measurement model, the standardised item loadings ranged from 0.595 to 0.814 (Figure 1).

**Figure 2:**
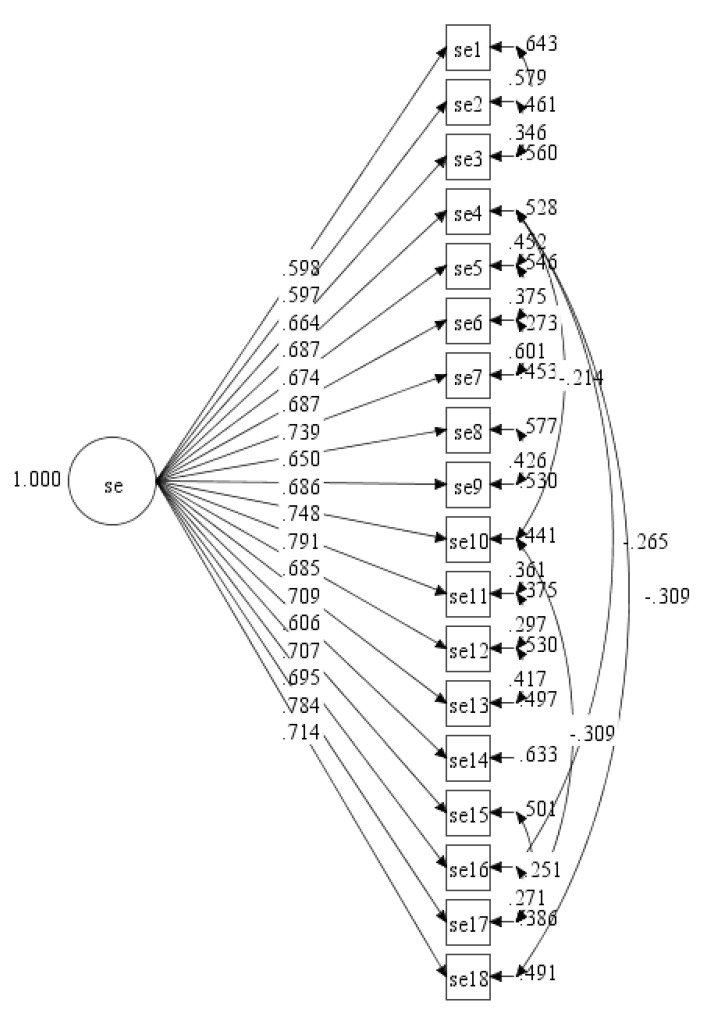
Final measurement model of ESE-M with single factor (se = self-efficacy).

Based on the initial CFA output, we found that the model fit indices of the initial model were not within the acceptable values as shown in Table 2. A further investigation was carried out in order to improve the model fit indices values. Several correlations between items’ residuals were added into the model iteratively, starting with the highest value of MI to the lower value of MI until the model fits the data. A final model with the accepted value of goodness of fit indices was achieved after several re-specification models were done (see Table 1, Final model; see Figure 2). Based on the final single factor model, the CR value for the self-efficacy factor was 0.921. 

#### 3.2.2. ESE-M with Three Factors

**Figure 3:**
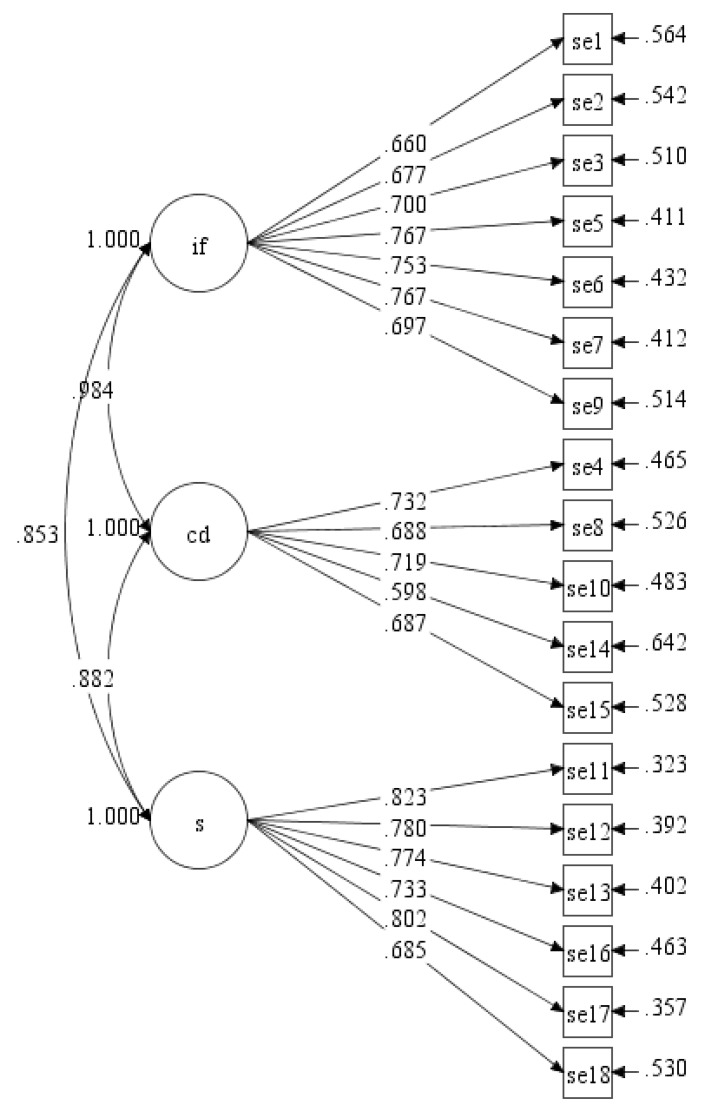
Initial measurement model of ESE-M with three factors (se = self-efficacy, if = internal feelings, cd = competing demands, s = situational).

Similar with the previous single factor model, the initial model of three factors showed all the factor loading of 18 items were above 0.40 (ranged between 0.598 and 0.823; see Figure 3). 

**Figure 4:**
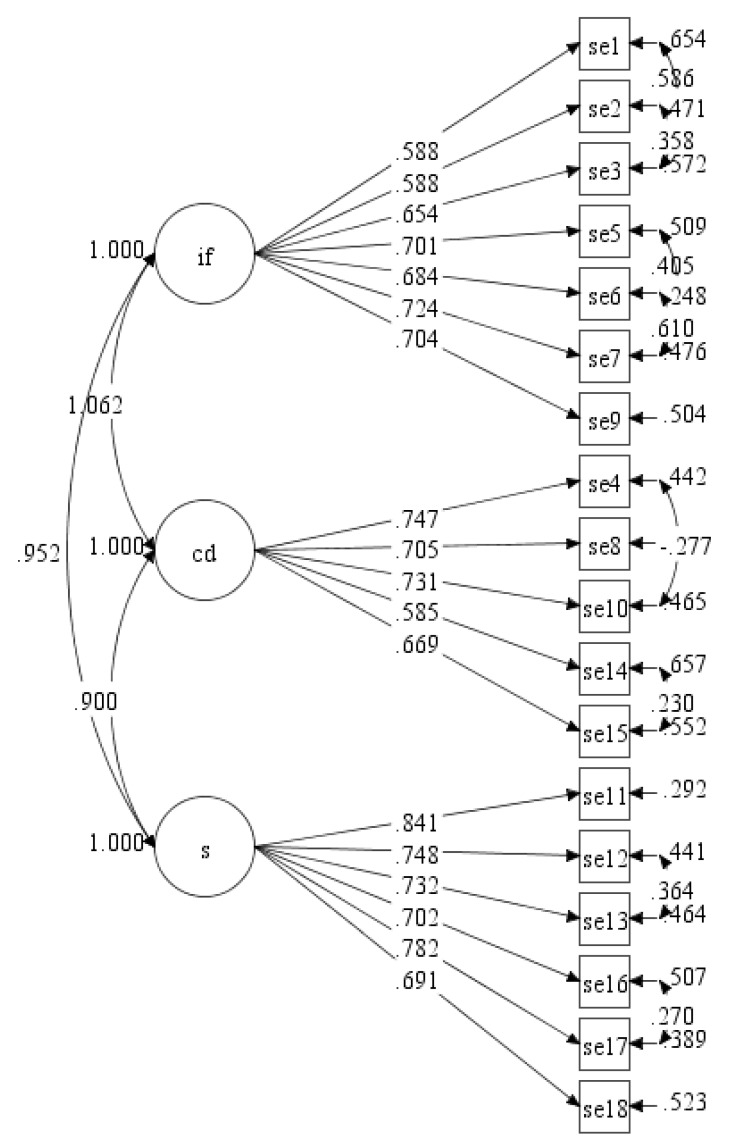
Final measurement model of ESE-M with three factors (se = self-efficacy, if = internal feelings, cd = competing demands, s = situational).

As for the model fit indices, the initial model of three factors model were not within the acceptable threshold value except for SRMR. Several model re-specifications were done iteratively by adding the correlation between items’ residuals within the same factor into the CFA analysis. After several re-specifications, the model showed improvement based on the model fit indices values (see Table 3, Final model; see Figure 4). However, the fit indices for CFI, TLI and RMSEA of the final model were still not within the acceptable threshold value. The MI value from the CFA result was inspected. However, no further improvement was suggested based on MI. Based on the final three factors model, the CR values for internal feelings, competing demands and situational were 0.762, 0.818 and 0.864, respectively.

Based on the CFA results, we compared the final model of both single factor and three factors models. It is noteworthy that the single factor model produced better goodness of fit indices than three factors model. 

## 4. Discussion

The present study aimed to assess the validity and reliability of the ESE-M scale among people with T2DM in HUSM, Malaysia, by performing CFA to find the model that fits the data. We identified the best fit measurement model for ESE-M that was deemed suitable to be used among people with T2DM in Malaysia. From the CFA result, no problematic items (factor loading < 0.40) were found, hence no items removal from the ESE-M. The final measurement model of ESE-M consists of 18 items and the reliability of scale was considered excellent. 

Exercise is one of the well-established regimens that can improve the condition of people with T2DM and their quality of life [32]. Furthermore, self-efficacy was an important factor that could promote PA among people with T2DM [33,34]. In Malaysia, the ESE-M has been used in general population especially young adults and school adolescents [14,35,36]. However, there is still limited study reporting the use of ESE-M and its validity among people with T2DM. Therefore, in the present study, we validated the ESE-M among people with T2DM and found the best ESE-M model structure (i.e., one factor or three factors) that is suitable for the T2DM population. 

Previous studies had reported the scale validation results of ESE in Malay [14] and English [37] versions. However, our study population was different from the study population reported in Sabo et al. [14] and Liu et al.’s [37] studies. Both studies assessed the validity of ESE scale in Malay [14] and English [37] versions among university undergraduate students. Liu et al. identified six problematic items, and they were removed iteratively [37]. They concluded three factors model of ESE with 12 items that provided good fit indices to their study population data. However, Sabo et al. found that either one factor or three factors structure measurement models of ESE-M fit their study population data well [14]. They recommended that both models are acceptable for use in measuring self-efficacy for exercise among university undergraduate students. Thus, the decision depends on whether the researchers want to interpret the self-efficacy scale score as a single score or as separate scores for the three factors (i.e., internal feelings, competing demands, situational). In the present study, we found that one-factor model outperformed the three factors model based on the fit indices provided in the CFA analysis. Thus, we recommend one-factor model of ESE-M as acceptable measure for use in evaluating exercise self-efficacy among people with T2DM. 

In comparison with the Dutch version [38] of the self-efficacy scale, by which T2DM patients were also the chosen sample, the CFA results showed a poor fit. Hence, modification on the model was taken, and five items were removed, leaving 13 items in the model. The remaining 13 items then produced adequate model fit indices values. In other regions, Iran, the Persian version [39] of the self-efficacy scale was also implied on diabetes patients with mean age of 46.94 years old. In contrast with the present study, the Persian version needed to remove one item in order to achieve the acceptable model fit indices values. There were some differences in the validation results between our present study and the studies conducted by Van der Heijden et al. [38] and Noroozi et al. [39]. This may vary due to the distinction of culture, exposure and socio-economic background between Europe region, Middle East regions and Malaysia.

We acknowledge that there were several limitations in the present study. The participants were recruited from a single hospital in Malaysia; thus, the generalisability external validity of the present study is limited. Although almost all of Malaysians apply the same culture, exposure, socio-economic and educational backgrounds, and other factors may lead to different understanding and interpretation of the questionnaire items. Insincerity and dishonesty could bring biases when a self-administered approach is applied. This may lead to the violation of the instrument reliability; however, before answering the questionnaire, participants were encouraged by the researcher to answer the questionnaire sincerely and honestly. They were also asked to not discuss with other patients while answering the questionnaire to avoid contamination. All these precautions were applied by the researcher as the best way to reduce bias. The data collection were taken in the area where the majority ethnic is Malay, thus, most of the participants were Malay. Therefore, future research should be conducted with better sampling method, which could balance participants between different ethnicities.

## 5. Conclusions

The results showed that the final model of single factor ESE-M is preferable to be adopted compared to the final model of three factors ESE-M. This indicates that the single factor ESE-M is more suitable to be adopted among Malaysians with T2DM. Thus, it can be beneficial in assessing individual self-efficacy of people with T2DM towards PA. Nonetheless, it is highly suggested for future studies to arrange better sampling method in order to collect a broader area of Malaysians with T2DM. More hospitals and clinics should be included in the data collection phase. A variety of socio-economic and educational backgrounds, rural and urban area, marital status and different layers of age also need to be concerned. Hence, results from the future study will be more comprehensive and could be generalised to all Malaysians with T2DM.

## Figures and Tables

**Table 1:** Demographic characteristics of type 2 diabetes mellitus (T2DM) patients in Hospital Universiti Sains Malaysia (HUSM) (*n* = 331).

Characteristics	Frequencies	Percentage	Mean (SD)
**Gender**			
Male	172	52.0%	
Female	159	48.0%	
**Age**			62.64 (0.56)
**Ethnicity**			
Malay	296	89.4%	
Chinese	25	7.6%	
Indian	6	1.8%	
Others	4	1.2%	
**Education background**			
Primary	87	26.3%	
Secondary	158	47.7%	
Diploma	60	18.1%	
Bachelor degree	26	7.9%	
**Occupation**			
Working/Business	92	27.9%	
Pensioners	139	42.0%	
Not working/Housewife	100	30.1%	
**Diabetic period**			
Less than 5 years	38	11.5%	
5 years or longer	32	9.7%	
10 years or longer	83	25.1%	
20 years or longer	178	53.8%	
**HbA1c** (mmol/mol)			76.90 (1.33)
**BMI** (kg/m^2^)			27.28 (0.28)

**Table 2:** Goodness of fit indices for measurement model of ESE-M single factor (initial and final models).

Model	CFI	TLI	SRMR	RMSEA (90% CI)	RMSEA (*p*-Value)
Initial model	0.760	0.728	0.071	0.114 (0.106, 0.122)	<0.001
Final model ^a^	0.952	0.938	0.044	0.054 (0.044, 0.065)	0.228

CFI = Comparative Fit Index, TLI = Tucker-Lewis Index, SRMR = Standardised Root Mean Square Residual, RMSEA = Root Mean Square Error of Approximation, CI = Confidence Interval. ^a^ Model with added correlated items’ residual.

**Table 3:** Goodness of fit indices for measurement model of ESE-M three factors (Initial and Final models).

Model	CFI	TLI	SRMR	RMSEA (90% CI)	RMSEA (*p*-Value)
Initial model	0.790	0.756	0.066	0.108 (0.100, 0.116)	<0.001
Final model ^a^	0.891	0.863	0.049	0.081 (0.072, 0.090)	<0.001

CFI = Comparative Fit Index, TLI = Tucker-Lewis Index, SRMR = Standardised Root Mean Square Residual, RMSEA = Root Mean Square Error of Approximation, CI = Confidence Interval. ^a^ Model with added correlated items’ residual.
